# Sugar-Free Milk

**Published:** 1901-07-06

**Authors:** 


### SUGAR-FREE MILK.

WE regret that in an article in last week's issue on "Sugar-free Milk" Dr. Hutchison's name was spelt Hutchinson.

